# Tomentosin a Sesquiterpene Lactone Induces Antiproliferative and Proapoptotic Effects in Human Burkitt Lymphoma by Deregulation of Anti- and Pro-Apoptotic Genes

**DOI:** 10.3390/life11111128

**Published:** 2021-10-23

**Authors:** Patrizia Virdis, Irene Marchesi, Francesco Paolo Fiorentino, Rossana Migheli, Luca Sanna, Valentina Bordoni, Giorgio Pintore, Grazia Galleri, Maria Rosaria Muroni, Luigi Bagella, Claudio Fozza, Maria Rosaria De Miglio, Luigi Podda

**Affiliations:** 1 Department of Medical, Surgical and Experimental Sciences, University of Sassari, 07100 Sassari, Italy; pavirdis@uniss.it (P.V.); rmigheli@uniss.it (R.M.); lusanna@uniss.it (L.S.); galleri@uniss.it (G.G.); mrmuroni@uniss.it (M.R.M.); podda@uniss.it (L.P.); 2 Kitos Biotech Srls, Porto Conte Ricerche, 07100 Sassari, Italy; imarchesi@kitosbiotech.org (I.M.); fpfiorentino@kitosbiotech.org (F.P.F.); 3 Department of Biomedical Sciences, University of Sassari, 07100 Sassari, Italy; v.bordoni@studenti.uniss.it (V.B.); lbagella@uniss.it (L.B.); 4 Department of Chemistry and Pharmacy, University of Sassari, 07100 Sassari, Italy; pintore@uniss.it; 5 Center for Biotechnology, Sbarro Institute for Cancer Research and Molecular Medicine, College of Science and Technology, Temple University, Philadelphia, PA 19122, USA

**Keywords:** tomentosin, Burkitt lymphoma, BCL2A1, CDKN1A, PMAIP1

## Abstract

(1) Tomentosin is the most representative sesquiterpene lactone extracted by *I. viscosa.* Recently, it has gained particular attention in therapeutic oncologic fields due to its anti-tumor properties. (2) In this study, the potential anticancer features of tomentosin were evaluated on human Burkitt’s lymphoma (BL) cell line, treated with increasing tomentosin concentration for cytotoxicity screening. (3) Our data showed that both cell cycle arrest and cell apoptosis induction are responsible of the antiproliferative effects of tomentosin and may end in the inhibition of BL cell viability. Moreover, a microarray gene expression profile was performed to assess differentially expressed genes contributing to tomentosin activity. Seventy-five genes deregulated by tomentosin have been identified. Downregulated genes are enriched in immune-system pathways, and PI3K/AKT and JAK/STAT pathways which favor proliferation and growth processes. Importantly, different deregulated genes identified in tomentosin-treated BL cells are prevalent in molecular pathways known to lead to cellular death, specifically by apoptosis. Tomentosin-treatment in BL cells induces the downregulation of antiapoptotic genes such as BCL2A1 and CDKN1A and upregulation of the proapoptotic PMAIP1 gene. (4) Overall, our results suggest that tomentosin could be taken into consideration as a potential natural product with limited toxicity and relevant anti-tumoral activity in the therapeutic options available to BL patients.

## 1. Introduction

Burkitt’s lymphoma is a highly aggressive B cell non-Hodgkin lymphoma (NHL), which originates from mature germinal or post germinal center B cells. BL is classified into three clinical entities, all of which are marked by chromosomal translocations such as t(8;14) (q24;q32) and its variants, t(2;8) (p12;q24.1) and t(8;22) (q24.1;q11.2), involving c-MYC oncogene. Properly, the constitutive activation of the c-MYC is followed by the abnormal transcriptional regulation of downstream genes, resulting in cellular transformation, inhibition of cell cycle checkpoints, and resistance to apoptosis [1,2]. Recently, next generation sequencing analysis has identified numerous somatic mutations in patients with BL, with the highest frequency for transcription factor TCF3 or its negative regulator ID3 [3]. BL accounts for less than 5% of lymphomas in adults. It also constitutes 40% of all childhood NHL, showing significant differences in clinical outcomes, with adult patients’ prognosis being extremely poor [4]. Moreover, over 70% of newly diagnosed patients display a stage III or IV disease at diagnosis [5]. Polychemotherapy plus immunotherapy at high “dose intensity” represents the most widely used approach for the different BL variants, considering the high biological aggressiveness of the disease [6,7]. In spite of the advances in clinical management of BL patients, many challenges persist above all in the context of treatment of immunocompromised patients, elderly individuals and patients with relapsed/refractory disease [8]. These data support the conviction that more effective or synergistic therapeutic approaches undoubtedly represent an unmet clinical need. Recently, Inula viscosa extracts revealed a potent anti-lymphoma activity, causing cell proliferation arrest and cell apoptosis induction in Raji cell line, and suggesting the involvement of cell cycle- and apoptosis-related genes known to be deregulated in BL cells [9]. A growing body of evidence shows that herbal medicines such as *Inula viscosa* (L.) Aiton consists of a multitude of sesquiterpene lactones (SL), representing excellent biologically active natural products with powerful therapeutic effects, which could be used in the treatment of several diseases. Recent works have displayed the potential anticancer effects of sesquiterpene lactones inducing antiproliferative effects in human cancer cell lines [10,11,12,13]. Xiang et al. have isolated five SL exhibiting remarkable cytotoxicity and inducing apoptosis associated with cleaved procaspase-3 and PARP against HEp2, SGC-7901 and HCT116 human cancer cell lines [14]. Interestingly, Yang et al. showed that tomentosin, a sesquiterpene lactone extracted by Inula viscosa, induces antiproliferative and proapoptotic events in leukemia cancer cells through the inhibition of mTOR/PI3K/AKT pathway [15].

This study aims to analyze the potential antitumoral activity on the human Raji cell line of the major representative sesquiterpene lactone extracted by *I. viscosa*, such as tomentosin. Furthermore, we tried to highlight possible insights into the molecular mechanisms involved in tomentosin anticancer properties, through an array-based gene expression analysis, thus potentially contributing to the identification of novel molecular markers and to an advancement in the therapeutic scenario of BL.

## 2. Materials and Methods

### 2.1. Cell Culture

Raji cells (ATCCCL-86, passage 11–25) were cultured as previously described [9]. Normal fibroblast cell line (MRC5) was obtained from IRCCS University Hospital San Martino-IST National Institute for Cancer Research (Genova, Italy) and cultured in DMEM high glucose (Euroclone) supplemented with 10% FBS (Euroclone), 2 mM L-Glutamine (Merck, Darmstadt, Germany), Non-essential amino acids, Sodium Pyruvate 1 mM (Merck), and Antibiotic/Antimycotic Solution. Cells were maintained at 37 °C in a 95% humidified atmosphere and 5% CO_2_ in T25 filtered flasks.

### 2.2. Cytotoxicity Assay

First, 7000 Raji cells or 1600 MRC5 cells were seeded in each well of a 384-multiwell microplate in a final volume of 25 µL. The day after, cells were treated with 50 µM, 25 µM, 12.5 µM, 6.25 µM, 3.125 µM, 1.56 µM or 0.75 µM Tomentosin (Mcule.com P-25953579) or Cisplatin (Merck C2210000), or with DMSO at 0.5% (vehicle) at 37 °C. After 48 h, cell viability was determined by CellTox Green Cytotoxicity (Promega Corporation, Madison, WI, USA G8731) and CellTiter-Glo (Promega G7571) assays using sequential multiplex protocol following manufacturer’s instructions. CellTox Green Dye was added to samples at the same time of treatment. Green fluorescence intensity (GFI) was quantified with Cytation 5 (BioTek, Winooski, VT, USA), covering the entire area of each well. Subsequently, CellTiter-Glo luminescent cell viability assay was performed, and relative luminescence units (RLU) were quantified with GloMax Discover (Promega). The half maximal inhibitory concentration values were calculated using R software version 3.2.2. (loess/approx functions), [16] given 100 to RLU observed in samples treated with vehicle solution and 0 to samples without cells (absolute IC50). The IC50 calculation was performed by using drug concentrations adjusted on compound purity (0.93× tomentosin). All assays were performed in triplicate.

### 2.3. Apoptotic Assay

Then, 10,000 cells were seeded in a 384-multiwell microplate in 20 μL per well. The day after, cells were treated with 50 µM, 25 µM, or 12.5 µM Tomentosin, or with DMSO 0.1% (vehicle). After 24 h, Caspase-Glo 3/7 (Promega G8090), Caspase-Glo 8 (Promega G8200), or Caspase-Glo 9 (Promega G8210) assays were performed as per manufacturer’s instructions. Luminescence reading was carried out with GloMax Discover Microplate Reader (Promega). All assays were performed in triplicate.

### 2.4. Cell Cycle Analysis

Then, 1 × 10^6^ Raji cells were seeded in 5 mL in T25 flask. The day after, cells were treated with 50 µM or DMSO 0.5% (vehicle). Samples were collected after 6, 12, 18, or 24 h of treatment. Samples were washed in PBS, fixed with 70% ice-cold ethanol and incubated at −20° overnight. Fixed cells were washed in PBS and stained with 7-aminoactinomycin (7-AAD; Bioscience, San Diego, CA, USA). DNA content of at least 10,000 cells for sample was evaluated with BD FACS CANTO II (BD Biosciences-US, Mylan, Italy), and values are presented as mean ± standard deviation (SD) of the percentage of three independent experiments. Data were analyzed with R-Bioc Manager software (flowCore/flowViz package) version 1.56.0 (https://bioconductor.org/packages/flowViz/, accessed on 23 October 2021) [17].

### 2.5. Gene Expression Analysis

Next, 3 × 10^6^ Raji cells were seeded in 15 mL in T75 flask. The day after, cells were treated with 25 µM Tomentosin or DMSO 0.05% (vehicle). Samples were collected after 18 h of treatment. Three biological replicates were performed. Expression of 2559 cancer-related genes was assessed by high-throughput mRNA quantitation (HTG EdgeSeq Oncology Biomarker Panel, HTG Molecular Diagnostics, PMID: 30327311). Counts were normalized and analyzed using the R-Bioc Manager software (DESeq2 package) version 3.0 [18]. Genes with fold change >1.5 or <0.66 in treated samples and false discovery rate (FDR) < 0.05 were considered as differentially expressed genes (DEGs). For Gene Set Enrichment Analysis (GSEA), normalized counts were uploaded into the GSEA-Broad Institute website (PMID: 16199517). 2257 gene sets from the Gene Ontology Biological Processes database (c5.bp.v7.1.symbols.gmt) were used as a library for analysis (PMID: 21546393). The statistical significance (nominal *p* value) of the normalized enrichment score (NES) was estimated by running 1000 gene set permutations.

### 2.6. Functional Classification and Pathway Analysis of DEGs

KEGG pathway analysis were executed to survey biological functions of all DEGs identified by the online software ToppCluster (https://toppcluster.cchmc.org/, accessed on 23 October 2021). Terms with FDR-corrected enrichment *p*-values <  0.05 were considered. While the interaction between altered genes and their matching terms were visualized by the Cytoscape program version 3.8.2 (http://www.cytoscape.org, accessed on 23 October 2021).

### 2.7. Statistical Data Analysis

R software, version 3.2.2 [16] was used to perform statistical analysis. All data are presented as mean ± SD from experiments in triplicate, and statistical significance between cell lines treated and untreated cells was set at *p* values < 0.05. Data analysis was performed by Kruskal and Wally test for the statistical analysis of cell viability and caspase activity, two-way ANOVA followed by Tukey’s post hoc test were applied for the statistical analysis of cell cycle.

## 3. Results

### 3.1. Effects of Tomentosin on Cell Viability in Human Burkitt Lymphoma Cells

To evaluate whether tomentosin could decrease the survival of human BL cells, we treated human BL and MRC5 cell lines (control) with increasing concentrations of the drug. Specifically, the cell lines were treated with tomentosin and cisplatin (50 µM, 25 µM, 12.5 µM, 6.25 µM, 3.125 µM, 1.56 µM, 0.75 µM) for 48 h and cell viability was determined. The IC50 values for each cell line were shown in Table 1.

**Figure 1:**
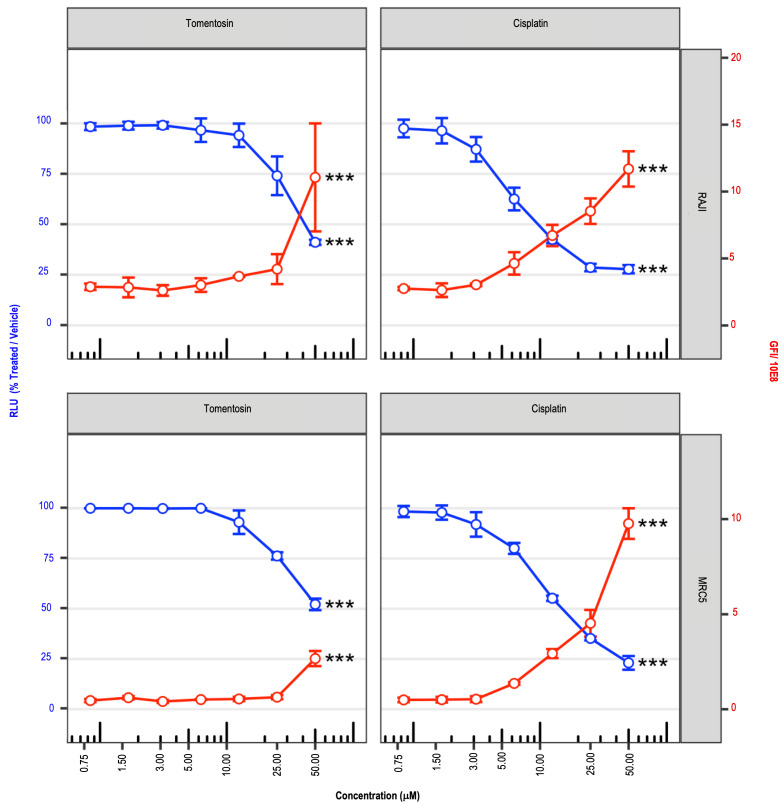
Cell viability reduction after tomentosin treatment. Cell viability scatterplots were obtained after 48 h of treatment with tomentosin or cisplatin at 50 µM, 25 µM, 12.5 µM, 6.25 µM, 3.12 µM, 1.56 µM or 0.8 µM. Relative luminescence units (RLU), obtained by addition of CellTiter-Glo reagent and representative of the number of living cells, were normalized to samples treated with vehicle solution (left y axis, blue lines and dots). The total, obtained by addition of CellTox green dye and representative of the number of dead cells, was divided by 10E8 (right y axis, red lines and dots). Mean ± SD values are shown. Kruskal-Wallis test was applied to test whether RLU values or GFI values obtained in treated and untreated samples originate from the same distribution. *** *p*-value < 0.010.

Figure 1 shows the effects of dose-dependent inhibitory growth activity of the tomentosin on cell viability at 48 h after treatments (blue lines). In details, it has been observed that tomentosin treatment did not produce changes in cell viability at low concentrations (in the range from 0.75 μM to 10.0 μM), while Raji cell viability dramatically decreased during tomentosin treatment, inducing cytotoxic effects beginning from 25.0 μM, and growing with the highest concentration. We therefore concluded that tomentosin exerts preferential antiproliferative activity towards the Raji cell line compared to normal fibroblast.

As shown by a red line in Figure 1, the proportional increase of cell death by tomentosin treatment in cell Raji agreed with the proportional decrease in cell viability following the increase of tomentosin concentrations.

### 3.2. Effects of Tomentosin on Apoptotic Cellular Processes in Human Burkitt Lymphoma Cells

Human BL and MCR5 cell lines were treated with tomentosin at concentrations of 12.5 µM, 25.0 µM, 50.0 µM, for 24 h to establish whether the cytotoxic effects of tomentosin depends on its ability to activate the apoptotic process.

**Figure 2:**
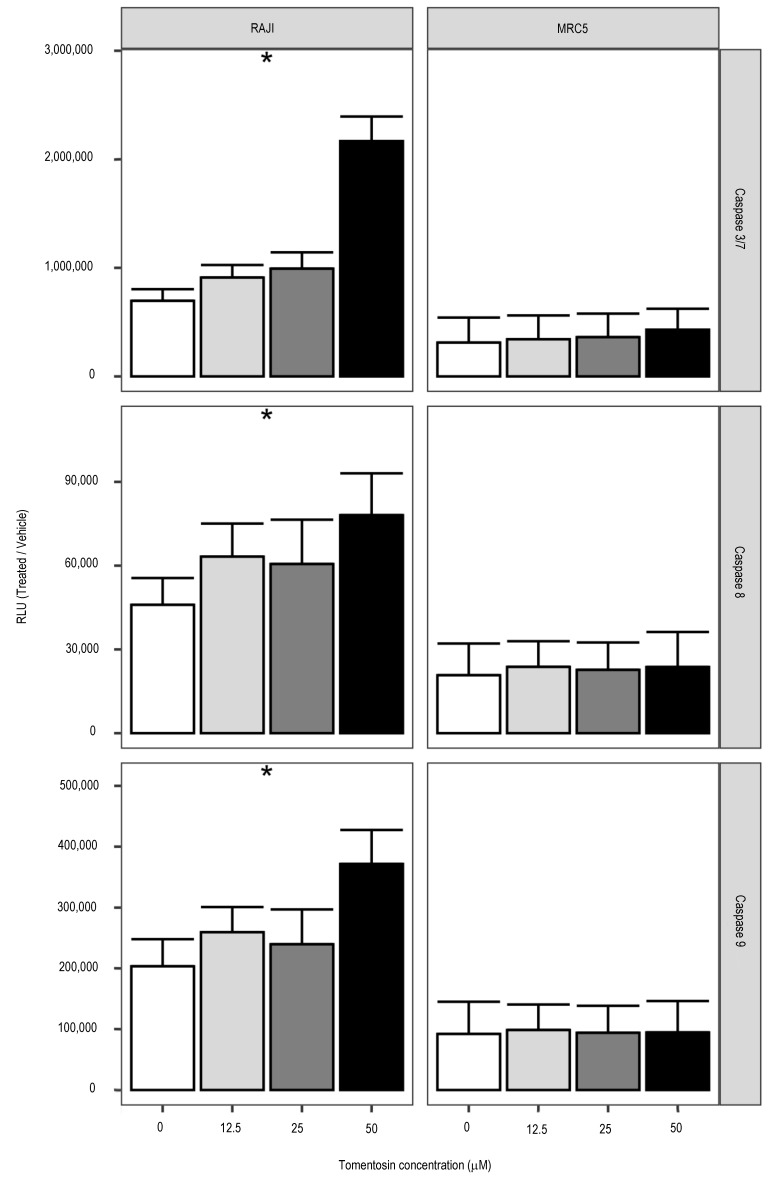
Increased caspase activities by tomentosin treatment. Histograms showing caspases 3/7, 8 or 9 cell-based enzymatic activity after 24 h of treatment with tomentosin at 12.5, 25 and 50 uM. Caspase activity in cells treated with vehicle solution is indicated as concentration zero. Mean ± SD values are shown. Kruskal-Wallis test was applied to test whether RLU values obtained in treated and untreated samples originate from the same distribution. * *p*-value < 0.05.

The enzymatic activities of caspase-3/7 (main effector caspases) and caspase-8 and 9 (initiator caspases) were measured using the Caspase-Glo 3/7, 8 and 9 assay. The RLUs revealed that tomentosin treatment increases the effector caspase activity (*p* < 0.05), such as those of caspase-8 and caspase-9 (*p* < 0.05), which are representative as initiator caspases in the apoptotic death receptor-mediated and mitochondrial pathways (Figure 2). Our findings suggest that tomentosin promotes apoptosis-induced death on Raji cells, facilitated by both the death receptor and the mitochondrial pathways activation.

### 3.3. Effects of Tomentosin on Cell Cycle in Human Burkitt Lymphoma Cells

**Figure 3:**
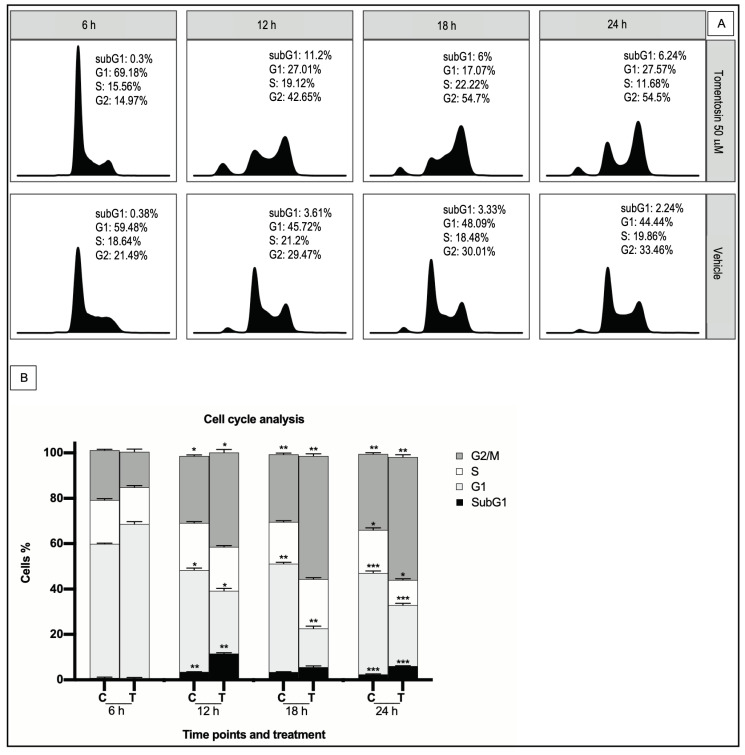
Effects of tomentosin on cell cycle progression in the Raji cell line. (**A**) Histograms show cell cycle profiles of tomentosin-treated Raji cells. X axis and y axis correspond to DNA content and fraction of events, respectively. (**B**) Raji cells treated with tomentosin 50 µM or vehicle solution (DMSO 0.05%) for 6, 12, 18, and 24 h have been blocked in the G2/M phase. Two-way ANOVA and Tukey’s post hoc test were applied for the statistical analysis of the cell cycle. The bars represent the mean and the standard deviation of the percentage of three independent experiments. Tomentosin treated cells vs control non treated, * *p* ≤ 0.0431; ** *p* ≤ 0.0046 and *** *p* ≤ 0.0007.

We performed DNA cell cycle analysis by flow cytometry in Raji cells treated with tomentosin or vehicle to analyze whether the anti-proliferative effect of tomentosin on BL cells was associated with cell cycle deregulation. Incubation with 50 µM tomentosin for 6, 12, 18 and 24 h showed a decrease of cell number in S-phase at 24 h (11.68% vs. 19.86% in control, *p* ≤ 0.0007) and an intense increase of cell number in G2/M-phase, which starts at 12 h (at 24 h: 54.5% vs. 33.46% in control, *p* ≤ 0.0431), accompanied by a corresponding decrease in the proportion of cells in G0/G1 (at 24 h: 27.57% vs. 44.44% in control, *p* ≤ 0.0046) (Figure 3). Our data demonstrates that the tomentosin treatment blocks Raji cells in G2/M phase, suggesting that both cell cycle arrest and cell apoptosis could explain the anti-proliferative effects of tomentosin.

### 3.4. Effects of Tomentosin on Gene Expression Profiling in Human Burkitt Lymphoma Cells

**Figure 4:**
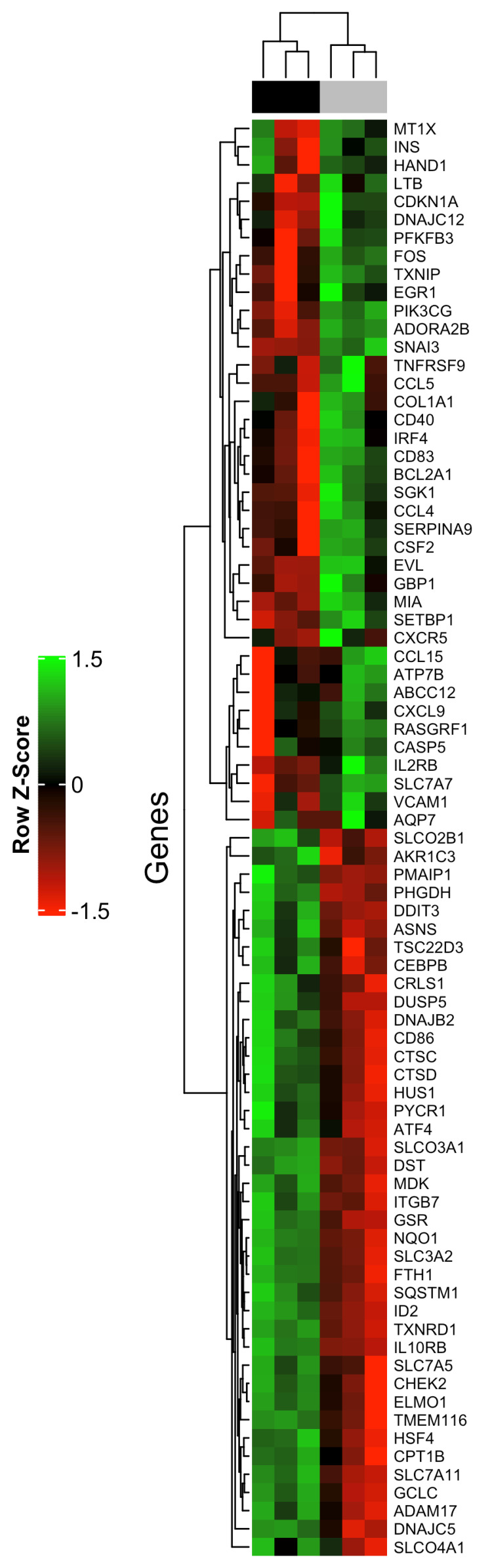
A 65-gene expression signature reveals changes between Raji cells treated and untreated with tomentosin. Unsupervised hierarchical clustering analysis of Raji cells treated and untreated with tomentosin was performed using 65 differentially expressed genes. Dendrograms of clustering analysis for samples and genes are displayed on the top and left, respectively. The relative up and down regulation of gene is indicated by red and green, respectively. Upper colour bar represents sample classes, black represents treated sample group (18 h treatment, 25 µM), grey represents untreated sample group (cells treated with vehicle solution).

The HTG EdgeSeq Oncology Biomarker Panel was used to analyze the expression of 2559 cancer-related genes by high-throughput mRNA quantitation analysis, to identify differentially expressed genes contributing to tomentosin effects on Raji cells and identify its possible mechanisms of action. Sixty-five genes were shown to be differentially expressed by comparing global gene expression between Raji cells treated with 25 µM tomentosin or untreated (vehicle). From 65 DEGs identified 42 genes were overexpressed and 23 genes were downregulated (Table S1). An unsupervised hierarchical clustering analysis of the 65 DEGs allowed to clearly separate tomentosin-treated Raji cells versus untreated cells (Figure 4).

**Figure 5:**
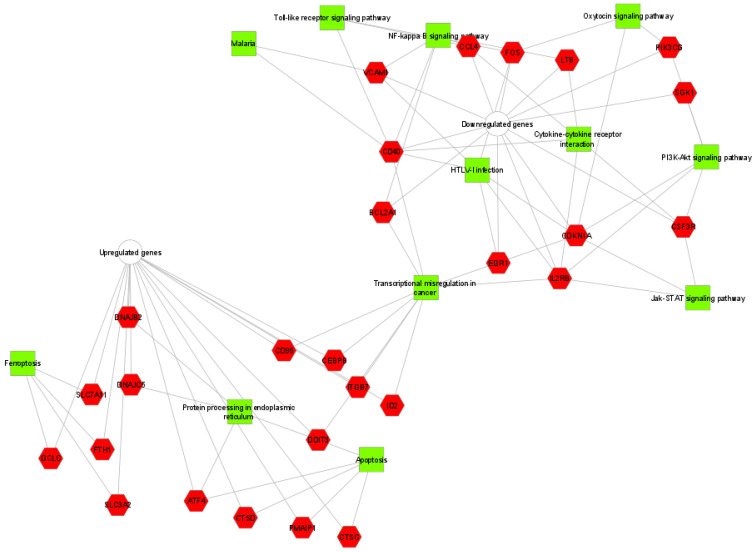
Human Burkitt lymphoma interactome network developed using Cytoscape. The 65 differentially expressed genes identified were first submitted to KEGG pathway enrichment analysis using ToppCluster and then displayed using Cytoscape.

A functional enrichment analysis was performed to analyze the biological role of the 65 DEGs deregulated by tomentosin treatment in Raji cells. We identified that the upregulated ASNS, ATF4, DDIT3 genes in tomentosin-treated Raji are involved in the biological process defined ER nucleus signaling pathway. Additionally, the significant identified DEGs were enriched in proteins that have key roles in cellular pathways such as NF-kappa B, PI3K/AKT, and JAK/STAT signaling pathway, transcriptional mis-regulation in cancer, apoptosis, ferroptosis, toll-like receptor signaling pathway, cytokine-cytokine receptor interaction, etc. (Table S2, Figure 5).

## 4. Discussion

Sesquiterpene lactones represent the most abundant and globally distributed groups of plant-derived bioactive compounds [14,19,20]. Our previous study, focused on the screening of antineoplastic activities of natural products, showing that *I. viscosa* extract exercises powerful antiproliferative and cytotoxic activities on human BL, showing a dose- and time-dependent decrease in cell viability, obtained by cell cycle arrest in the G2/M phase and an increase in cell apoptosis. The molecular mechanisms underlying such an antineoplastic activity are based on targeting and downregulation of genes involved in cell cycle and apoptosis cell processes [9].

In this study, we focused on investigating if tomentosin, the most representative natural potent bioactive compound extracted from the *I. viscosa* plant, might be responsible for the bioactivity of *I. viscosa* extract in the same tumor.

Our study showed that tomentosin exerts a powerful antiproliferative and cytotoxic activity against human BL cell line. Especially, the treatment of this tumor with increasing concentrations of tomentosin induces dose-dependent decrease in cell viability, followed by a reduction in cell proliferation due to cell cycle arrest in the G2/M phase. Concurrently, the same concentrations of tomentosin did not induce cytotoxic effects in normal MRC5 cells, suggesting a selective activity of the tomentosin against BL cells. Additionally, a dose-dependent increase in cell apoptosis was observed, by activating both the death receptor and the mitochondrial pathways on Raji cells. These findings suggest that both cell cycle arrest and cell apoptosis could be the main mechanisms explaining the anti-proliferative effects of tomentosin, resulting in the inhibition of Raji cell viability. As support, Yang et al. demonstrated that tomentosin induces apoptosis in human leukemia cells by caspase-facilitated pro-apoptotic pathway, and inhibition of the NF-κB-stimulated Bcl-2 [15]. Furthermore, tomentosin is identified as one of the most beneficial natural therapeutic agents against various solid tumors [10,11,12,13].

In order to assess the molecular mechanisms involved in tomentosin anti-neoplastic activity, a high-throughput mRNA expression profiling on 2559 cancer-related genes was performed. We identified 65 DEGs showing different activities in human BL cells treated or untreated with tomentosin. Deregulation of various biological pathways is a hallmark in tumor biology. Our data emphasize that a great number of DEGs deregulated by tomentosin treatment influence functional cell processes, which is absolutely crucial to determinate and support the BL phenotype.

In more detail, we determined that overexpressed genes identified in tomentosin-treated BL cells are prevalent in pathways known to lead to cellular death, specifically via apoptosis and ferroptosis processes, and protein processing in endoplasmic reticulum. Genes down-regulated in BL cells are known to manage pathways that regulate growth, differentiation, migration and also apoptotic cell processes, such as PI3K-Akt and JAK/STAT signaling pathway. Downregulated genes are also involved in immune-system processes, as well as NF-kappa B pathways, toll-like receptor signaling, cytokine-cytokine receptor interaction (CCL4, CD40, LTB, VCAM1, CSF3R, IL2RB and FOS genes).

Interestingly, our data identify the induction of apoptosis processes through different molecular pathways. The pro-apoptotic PMAIP1 (NOXA) gene was upregulated in human BL cells treated with tomentosin. Yee et al. demonstrated that latent EBV in B cells inhibits apoptosis induced by ionomycin and/or staurosporine in BL-derived B cells, modifying the NOXA expression levels [21]. Moreover, upregulation of DNAJB2 and DNAJC5 genes were identified in human BL cells treated with tomentosin, which control protein folding and misfolded protein degradation, intracellular trafficking, regulating cellular signaling pathways and immune responses [22,23]. Additionally, ATF4 and DDIT3 overexpression was observed, whose proteins are enriched in protein processing in the endoplasmic reticulum pathway. In neoplastic disease, the chronic activation of ER stress response can trigger apoptotic signals, leading to the expression of downstream signaling, which damages the target cells [24]. It should be suggested that the ATF4 and DDIT3 overexpression induced by tomentosin in human BL may be responsible for the activation of the unfolded protein response PERK/eIF2a/ATF4/DDIT3 pathway. The tomentosin-induced overexpression of the transcription factor ATF4, which further activates the proapoptotic CHOP (DDIT3), also activated by the drug, suggests that in presence of tomentosin the protective cell mechanisms activated by the unfolded protein response signaling is not sufficient to restore normal ER function and cells can be induced to apoptosis.

Interestingly, tomentosin treatment in human BL induces downregulation of BCL2A1 gene, which is a highly regulated NF-kB target gene, involved in major pro-survival functions. Physiologically, BCL2A1 is mainly expressed in the hematopoietic system, facilitating survival of selected leukocyte subgroups and inflammation. Besides, BCL2A1 is overexpressed in various neoplastic cells, including hematological malignancies [25,26,27,28,29] and solid tumors [30,31,32], and is known to contribute to tumor progression [33,34,35]. Considering that the main BCL2A1 function is to control the release of cytochrome c from mitochondria in the intrinsic apoptotic pathway, it may be an attractive target for anti-cancer therapies. The gene expression profile performed on tomentosin-treated human BL shows as tomentosin deregulates the BCL2A1 gene expression and could promote mitochondrial apoptosis pathway in BL cells.

The CDKN1A (p21) protein seems to perform contrasting tumor suppressor or oncogenic functions in neoplastic cells. Recently, a growing amount of evidence indicates their oncogenic properties, mechanistically based on protection from apoptosis [36]. Accordingly, the accumulation of cytoplasmic p21, stimulated by AKT- or IKKβ-dependent pathways, suppresses programmed cell death [37]. Interestingly, studies on tumor development in mice have demonstrated that the deletion of CDKN1A inhibits lymphoma [38,39]. In agreement with the CDKN1A oncogenic activity, lymphomas arising in CDKN1A-deficient mice demonstrate a high rate of apoptosis [40]. Our data show the CDKN1A downregulation induced in human BL from tomentosin and suggest that this pathway represent a mechanism to induce neoplastic cell apoptosis.

The pharmacologic effects of Tomentosin on BL progression are exclusively based on gene expression profiling and enrichment analysis obtained in in vitro experiments, representing a limitation of our work. Based on previous results, our future investigations will include in vivo experiments to analyze the molecular mechanisms by which tomentosin induce its pharmacologic effects in human BL.

## 5. Conclusions

In summary, our results demonstrate that tomentosin has a potent anti-tumoral activity on human BL cells mediated by cell proliferation inhibition and cell apoptosis induction. Furthermore, in a gene expression profiling analysis we investigated the molecular mechanisms responsible for tomentosin effects on human BL. Tomentosin induces the downregulation of genes enriched in immune-system pathways, as well as pathways which favor proliferation and cellular growth processes. Importantly, we demonstrated that different deregulated genes identified in tomentosin-treated BL cells are prevalent in molecular pathways known to lead to cellular death, especially by apoptosis. Our results further suggest that tomentosin could be taken into consideration as a potential natural product with limited toxicity and relevant anti-tumoral activity among the therapeutic options available for BL patients.

## Figures and Tables

**Table 1:** IC50 values of Tomentosin and Cisplatin after 48 h of treatment.

Cell Line	Treatment	IC50	Confidence Interval 95%
Raji	Tomentosin	42.62 µM	39.53–46.47 µM
MRC5	Tomentosin	>50.00 µM	—
Raji	Cispaltin	9.43 µM	8.23–11.21 µM
MRC5	Cispaltin	14.77 µM	13.1–16.48 µM

## Data Availability

All data generated or analyzed during this study are included in this published article.

## Supplementary Materials

The following are available online at https://www.mdpi.com/article/10.3390/life11111128/s1, Table S1: List of differentially deregulated genes targeted by Tomentosin in Raji cell line, Table S2: The enriched pathways of differentially expressed genes targeted by Tomentosin in RAJI cell line.

## Abbreviations

BL: Burkitt’s lymphoma, NHL: non-Hodgkin lymphoma, SL: sesquiterpene lactones, MRC5: normal fibroblast cell line, GFI: green fluorescence intensity, RLU: relative luminescence units, 7-AAD: 7-aminoactinomycin, SD: standard deviation, FDR false discovery rate, DEGs: differentially expressed genes, GSEA: gene Set Enrichment Analysis, NES: normalized enrichment score.

## References

[1] Casulo C., Friedberg J.W. (2018). Burkitt lymphoma- a rare but challenging lymphoma. Best Pract. Res. Clin. Haematol..

[2] Dang C.V., O’Donnell K.A., Juopperi T. (2005). The great MYC escape in tumorigenesis. Cancer Cell.

[3] Dunleavy K., Little R.F., Wilson W.H. (2016). Update on Burkitt Lymphoma. Hematol. Oncol. Clin. N. Am..

[4] Bishop P.C., Rao V.K., Wilson W.H. (2000). Burkitt’s lymphoma: Molecular pathogenesis and treatment. Cancer Investig..

[5] Jaffe E.S. (2009). The 2008 WHO classification of lymphomas: Implications for clinical practice and translational research. Hematol. Am. Soc. Hematol. Educ. Program.

[6] Saleh K., Michot J.M., Camara-Clayette V., Vassetsky Y., Ribrag V. (2020). Burkitt and Burkitt-Like Lymphomas: A Systematic Review. Curr. Oncol. Rep..

[7] Minard-Colin V., Aupérin A., Pillon M., Burke G.A.A., Barkauskas D.A., Wheatley K., Delgado R.F., Alexander S., Uyttebroeck A., Bollard C.M. (2020). Rituximab for High-Risk, Mature B-Cell Non-Hodgkin’s Lymphoma in Children. N. Engl. J. Med..

[8] Molyneux E.M., Rochford R., Griffin B., Newton R., Jackson G., Menon G., Harrison C.J., Israels T., Bailey S. (2012). Burkitt’s lymphoma. Lancet.

[9] Virdis P., Migheli R., Galleri G., Fancello S., Cadoni M.P.L., Pintore G., Petretto G.L., Marchesi I., Fiorentino F.P., di Francesco A. (2020). Antiproliferative and proapoptotic effects of Inula viscosa extract on Burkitt lymphoma cell line. Tumor Biol..

[10] Rozenblat S., Grossman S., Bergman M., Gottlieb H., Cohen Y., Dovrat S. (2008). Induction of G2/M arrest and apoptosis by sesquiterpene lactones in human melanoma cell lines. Biochem. Pharmacol..

[11] Merghoub N., El Btaouri H., Benbacer L., Gmouh S., Trentesaux C., Brassart B., Attaleb M., Madoulet C., Wenner T., Amzazi S. (2017). Tomentosin Induces Telomere Shortening and Caspase-Dependant Apoptosis in Cervical Cancer Cells. J. Cell. Biochem..

[12] Lee C.M., Lee J., Nam M.J., Choi Y.S., Park S.H. (2019). Tomentosin displays anti-carcinogenic effect in human osteosarcoma MG-63 cells via the induction of intracellular reactive oxygen species. Int. J. Mol. Sci..

[13] Yang H., Zhao H., Dong X., Yang Z., Chang W. (2020). Tomentosin induces apoptotic pathway by blocking inflammatory mediators via modulation of cell proteins in AGS gastric cancer cell line. J. Biochem. Mol. Toxicol..

[14] Xiang P., Guo X., Han Y.-Y., Gao J.-M., Tang J.-J. (2016). Cytotoxic and Pro-apoptotic Activities of Sesquiterpene Lactones from Inula Britannica. Nat. Prod. Commun..

[15] Yang L., Xie J., Almoallim H.S., Alharbi S.A., Chen Y. (2021). Tomentosin inhibits cell proliferation and induces apoptosis in MOLT-4 leukemia cancer cells through the inhibition of mTOR/PI3K/Akt signaling pathway. J. Biochem. Mol. Toxicol..

[16] Ihaka R., Gentleman R. (1996). R: A Language for Data Analysis and Graphics. J. Comput. Graph. Stat..

[17] Ellis B., Gentleman R., Hahne F., Le Meur N., Sarkar D., Jiang M. (2021). flowViz: Visualization for Flow Cytometry. R Package Version 1.56.0. https://bioconductor.org/packages/release/bioc/html/flowViz.html.

[18] Love M.I., Huber W., Anders S. (2014). Moderated estimation of fold change and dispersion for RNA-seq data with DESeq2. Genome Biol..

[19] Wang G.W., Qin J.J., Cheng X.R., Shen Y.H., Shan L., Jin H.Z., Zhang W.D. (2014). Inula sesquiterpenoids: Struc-tural diversity, cytotoxicity and anti-tumor activity. Expert Opin. Investig. Drugs.

[20] Quintana J., Estévez F. (2019). Recent Advances on Cytotoxic Sesquiterpene Lactones. Curr. Pharm. Des..

[21] Yee J., White R.E., Anderton E., Allday M.J. (2011). Latent Epstein-Barr virus can inhibit apoptosis in B cells by blocking the induction of NOXA expression. PLoS ONE.

[22] Crawley J.B., Williams L.M., Mander T., Brennan F.M., Foxwell B.M.J. (1996). Interleukin-10 stimulation of phosphatidylinositol 3-kinase and p70 S6 kinase is required for the proliferative but not the antiinflammatory effects of the cytokine. J. Biol. Chem..

[23] Zhou J.-H., Broussard S.R., Strle K., Freund G.G., Johnson R.W., Dantzer R., Kelley K.W. (2001). IL-10 Inhibits Apoptosis of Promyeloid Cells by Activating Insulin Receptor Substrate-2 and Phosphatidylinositol 3′-Kinase. J. Immunol..

[24] Tsao C.C., Geisen C., Abraham R.T. (2004). Interaction between human MCM7 and Rad17 proteins is required for replication checkpoint signaling. EMBO J..

[25] Nagy B., Lundán T., Larramendy M.L., Aalto Y., Zhu Y., Niini T., Edgren H., Ferrer A., Vilpo J., Elonen E. (2003). Abnormal expression of apoptosis-related genes in haematological malignancies: Overexpression of MYC is poor prognostic sign in mantle cell lymphoma. Br. J. Haematol..

[26] Feuerhake F., Kutok J.L., Monti S., Chen W., LaCasce A.S., Cattoretti G., Kurtin P., Pinkus G.S., De Levai L., Harris N.L. (2005). NFκB activity, function, and target-gene signatures in primary mediastinal large B-cell lymphoma and diffuse large B-cell lymphoma subtypes. Blood.

[27] Mahadevan D., Spier C., Della Croce K., Miller S., George B., Riley C., Warner S., Grogan T.M., Miller T.P. (2005). Transcript profiling in peripheral T-cell lymphoma, not otherwise specified, and diffuse large B-cell lymphoma identifies distinct tumor profile signatures. Mol. Cancer Ther..

[28] Piva R., Pellegrino E., Mattioli M., Agnelli L., Lombardi L., Boccalatte F., Costa G., Ruggeri B.A., Cheng M., Chiarle R. (2006). Functional validation of the anaplastic lymphoma kinase signature identifies CEBPB and BCl2A1 as critical target genes. J. Clin. Investig..

[29] Monti S., Savage K.J., Kutok J.L., Feuerhake F., Kurtin P., Mihm M., Wu B., Pasqualucci L., Neuberg D., Aguiar R.C.T. (2005). Molecular profiling of diffuse large B-cell lymphoma identifies robust subtypes including one characterized by host inflammatory response. Blood.

[30] Park I., Lee S., Whang D., Hong W., Choi S., Shin H., Choe T., Hong S. (1997). Expression of a novel Bcl-2 related gene, Bfl-1, in various human cancers and cancer cell lines. Anticancer. Res..

[31] Kathpalia V.P., Mussak E.N., Chow S.S., Lam P.H., Skelley N., Time M., Markelewicz R.J., Kanduc D., Lomas L., Xiang Z. (2006). Genome-wide transcriptional profiling in human squamous cell carcinoma of the skin identifies unique tumor-associated signatures. J. Dermatol..

[32] Saleh A., Zain R.B., Hussaini H., Ng F., Tanavde V., Hamid S., Chow A.T., Lim G.S., Abraham M.T., Teo S.H. (2010). Transcriptional profiling of oral squamous cell carcinoma using formalin-fixed paraffin-embedded samples. Oral Oncol..

[33] Yoon H.S., Hong S.H., Kang H.J., Ko B.K., Ahn S.H., Huh J.R. (2003). Bfl-1 Gene Expression in Breast Cancer: Its Relationship with other Prognostic Factors. J. Korean Med. Sci..

[34] Riker A.I., Enkemann S.A., Fodstad O., Liu S., Ren S., Morris C., Xi Y., Howell P., Metge B., Samant R.S. (2008). The gene expression profiles of primary and metastatic melanoma yields a transition point of tumor progression and metastasis. BMC Med. Genomics.

[35] Lee C.F., Ling Z.Q., Zhao T., Fang S.H., Chang W.C., Lee S.C., Lee K.R. (2009). Genomic-wide analysis of lymphatic metastasis-associated genes in human hepatocellular carcinoma. World J. Gastroenterol..

[36] Dotto G.P. (2000). p21(WAF1/Cip1): More than a break to the cell cycle?. Biochim. Biophys. Acta Rev. Cancer.

[37] Abbas T., Dutta A. (2009). p21 in cancer: Intricate networks and multiple activities. Nat. Rev. Cancer.

[38] De La Cueva E., García-Cao I., Herranz M., López P., García-Palencia P., Flores J.M., Serrano M., Fernández-Piqueras J., Martín-Caballero J. (2006). Tumorigenic activity of p21Waf1/Cip1 in thymic lymphoma. Oncogene.

[39] Martín-Caballero J., Flores J.M., García-Palencia P., Serrano M. (2001). Tumor Susceptibility of p21 Waf1/Cip1-deficient Mice 1. Cancer Res..

[40] Roninson I.B. (2002). Oncogenic functions of tumour suppressor p21Waf1/Cip1/Sdi1: Association with cell senescence and tumour-promoting activities of stromal fibroblasts. Cancer Lett..

